# Performance Research of Cement Concrete Pavements with a Lower Carbon Footprint

**DOI:** 10.3390/ma17133162

**Published:** 2024-06-27

**Authors:** Tomasz Rudnicki, Przemysław Stałowski

**Affiliations:** 1 Faculty of Civil Engineering and Geodesy, Military University of Technology, 2 Kaliskiego St, 00-908 Warsaw, Poland; 2 Faculty of Civil and Environmental Engineering and Architecture, Bydgoszcz University of Science and Technology, 85-796 Bydgoszcz, Poland; przemyslaw.stalowski@pbs.edu.pl

**Keywords:** road concrete, multi-component cements, reduced carbon footprint of concrete

## Abstract

The growing interest in the use of building materials with a reduced carbon footprint was the aim of this research assessing the impact of four different types of low-emission cements on the properties of cement concretes used for the construction of local roads. This research work attempted to verify the strength characteristics and assess the durability of such solutions, which used the commonly used CEM I 42.5 R pure clinker cement and three multi-component cements: CEM II/A-V 42.5 R, CEM III/A 42.5 N-LH/HSR/NA, and CEM V/A S-V 42.5 N-LH/HSR/NA. Cement was used in a constant amount of 360 kg/m^3^, sand of 0/2 mm, and granite aggregate fractions of 2/8 and 8/16 mm. This research was carried out in two areas: the first concerned strength tests and the second focused on the area of assessing the durability of concrete in terms of frost resistance F150, resistance to de-icing agents, water penetration under pressure, and an analysis of the air entrainment structure in concrete according to the PN EN 480-11 standard. Analyzing the obtained test results, it can be concluded that the highest compressive strength of more than 70 MPa was obtained for CEM III concrete, 68 MPa for CEM V concrete, and the lowest for CEM I cement after 90 days. After the durability tests, it was found that the smallest decrease in compressive strength after 150 freezing and thawing cycles was obtained for CEM III (−0.9%) and CEM V (−1.4%) concretes. The high durability of concrete is confirmed by water penetration tests under pressure, because for newly designed recipes using CEM II, CEM III, and CEM V, water penetration from 17 mm to 18 mm was achieved, which proves the very high tightness of the concrete. The assessment of the durability of low-emission cements was confirmed by tests of resistance to de-icing agents and the aeration structure performed under a microscope in accordance with the requirements of the PN-EN 480-11 standard. The obtained analysis results indicate the correct structure and minimal spacing of air bubbles in the concrete, which confirms and guarantees the durability of concrete intended for road construction. Concretes designed using CEM V cement are characterized by a carbon footprint reduction of 36%, and for the mixture based on CEM III, we even observed a decrease of 39% compared to traditional concrete. Concrete using CEM II, CEM III, and CEM V cements can be successfully used for the construction of local roads. Therefore, it is necessary to consider changing the requirements of the technical specifications recommended for roads in Poland.

## 1. Introduction

With the development of the road network in Poland, growing requirements of investors and road users are expected related to the use of materials that affect the possibility of reducing CO_2_ emissions [1,2,3,4]. The need to take action to reduce the carbon footprint by reducing the emissions of individual components of concrete as a building material is one of the challenges facing the construction industry in the context of climate protection, as reflected in the very ambitious CO_2_ emission reduction goals under the Green Deal Program announced by the European Commission in December 2019 [5,6].

The entire building materials industry in Europe will have to rise to this challenge by 2050. The European Green Deal assumes that over the next 10 years, Europe will be able to reduce CO_2_ emissions by 55% of the amount of CO_2_ emitted in 1990 and achieve full climate neutrality by 2050. The search for low-emission materials and production technologies must cover the entire supply chain because, in the case of modern, energy-efficient cement production technologies, the possibilities for reduction at this stage are significantly limited [7,8,9,10]. Additionally, reducing CO_2_ emissions from cement production is a particular challenge because only approximately 40% of direct emissions come from the combustion of fuels, while the remaining 60% of CO_2_ results from the decomposition of raw materials subjected to thermal treatment in a cement kiln [11]. 

Additionally, as in many other industrial processes, CO_2_ emissions resulting from electricity consumption must also be taken into account. Only the implementation of innovative technological and product solutions and a longer-term approach can bring significant effects in this area [12,13,14]. The most effective technology for reducing CO_2_ emissions from cement plants is the method of capturing CO_2_ and then storing it in geological structures or using it as a raw material in various technologies (Carbon Capture Usage/Storage—CCUS). Pilot research on this technology has been ongoing for several years, but currently in Poland, Holcim, a leader in environmental protection, has started work on this technology and plans to implement it on an industrial scale in 2027. 

An important tool in climate policy is the assessment of the carbon footprint of products, which requires an analysis of the entire product life cycle [15,16]. In the case of cement concrete, a life cycle analysis should be carried out for each stage, especially for the stage of demolition of the concrete pavement through recycling and its reinstallation, which constitutes the closure of the cycle and is in line with the circular economy strategy [16,17,18]. It is worth emphasizing the positive impact of CCP (Cement Concrete Pavements) on reducing the ambient temperature compared to asphalt pavements. Studies were conducted on the development of important climatic characteristics (maximum and average annual temperature and the frost index) of Central Europe (CE) used in the design of CCPs. The research clearly confirmed a significant increase in CE temperatures and the resulting changes in pavement stresses [19]. 

This idea assumes the optimization of the use of materials in pavement construction and the reuse of recycled materials after the end of pavement operation [20,21]. The reliability of the assessment of the carbon footprint of pavement concrete should also take into account an additional process that takes place during the use of concrete structures—carbonation, i.e., the process of absorbing CO_2_ by concrete. 

Analyzing the achievements in terms of reducing the carbon footprint, it should be noted that a significant reduction effect in CO_2_ emissions can be achieved by limiting the amount of Portland clinker in cement [1,14,17]. Traditional Portland cement without additives contains >90% Portland clinker and is mainly made of limestone. Producing 1 ton of clinker requires, on average, 3.7 GJ of heat and emits approximately 850 kg of CO_2_ [18]. Lower emissions in the case of reductions in the clinker/cement ratio (currently, in Poland, this indicator is 75%) results, on the one hand, from lower process emissions from the calcination of raw materials and, at the same time, from the lower heat demand for the production of clinker minerals [16,18]. On the other hand, the possibility of using mineral additives such as blast furnace slag, fly ash [8,22,23,24,25,26,27,28], and limestone, which partially replace clinker in cement, significantly reduces the carbon footprint, which was demonstrated in [11,29,30]. 

Another element enabling the reduction in the carbon footprint is the possibility of using alternative aggregates, especially local ones [9,30], which, due to shorter transport distances, can reduce CO_2_ emissions by up to 11%. The modification of the composition of cement concrete should also take into account the impact of the use of mineral additives that are industrial waste (stone dust from aggregate dedusting or additives such as fly ash and microsilica) [23,24,25,26,27,28]. Obtaining high-strength parameters of cement concrete can be achieved by using chemical admixtures that strongly reduce the amount of water and increase workability over time [31,32,33,34], and the use of air-entraining admixtures significantly increases the durability of concrete over time. 

An important element of maintaining the fluidity of road user traffic is the possibility of performing quick renovation works under traffic, which is described in detail in a previous article [35]. When performing a full life cycle analysis, the assessment of the durability of the designed solutions should be taken into account, especially the possibility of reusing the materials used in terms of recycling concrete [18]. The possibility of reusing cement concrete after recycling has been repeatedly confirmed in studies and even directly in the implementation of a concrete highway in Poland [20,21]. In this work, the authors attempted to use low-emission cements in cement concrete road surfaces in order to achieve the maximum reduction in the carbon footprint, which was confirmed by testing the frost resistance of F150 and analyzing the structure of concrete air entrainment.

## 2. Materials and Methods

In order to analyze the possibility of reducing the carbon footprint in concrete used to build road surfaces, the following types of cement were used in accordance with the PN-EN 197-1 standard [36]: CEM I 42.5 R, CEM II/A-V 42.5 R, CEM III/A 42.5 N-LH/HSR/NA, and CEM V/A S-V 42.5 N-LH/HSR/NA. In the requirements for the construction of road infrastructure in Poland, cements from the CEM I group are permitted for use, while the use of cements with mineral additives requires the investor to present not only an analysis of the reduction in the carbon footprint but also, above all, durability over time. Therefore, this publication presents comprehensive tests of strength characteristics after 28, 56, and 90 days, and more importantly, tests of resistance to frost and water penetration under pressure. In order to obtain the most comparable test results, granite aggregate in fractions of 2/8 and 8/16 with a density of 2.64 g/cm^3^ and water absorption of 0.80% and natural non-alkaline reactive sand were used in all designed recipes, with a density of fraction of 0/2 of 2.65 g/cm^3^. Due to the method of transport, installation, and compaction, a low *w*/*c* ratio of 0.4 was required. Two types of chemical admixtures were used in the form of a new-generation fluidizing admixture marked as SP in accordance with the PN EN 934-1 [37] and PN-EN 934-2 [38] standards (admixture with a high degree of water reduction with a density of 1.01 g/cm^3^) and a traditionally used air-entraining admixture. 

### 2.1. Analysis of the Properties and Microstructure of Cements

The physical and mechanical properties of the cements used are presented in Table 1.

The mineral additives used in individual cements comply with the EN 197-1 standard [36] and are as follows: CEM I 42.5 R 95% clinker, 5% secondary minerals, CEM II A-V 80–94% clinker, 6–20% silica fly ash, 5% secondary minerals, CEM III A 35–64% clinker, 36–65% blast furnace slag, 5% secondary minerals, CEM V A 40–64% clinker, 18–30% blast furnace slag, 18–30% silica fly ash, and 5% secondary minerals.

**Figure 1:**
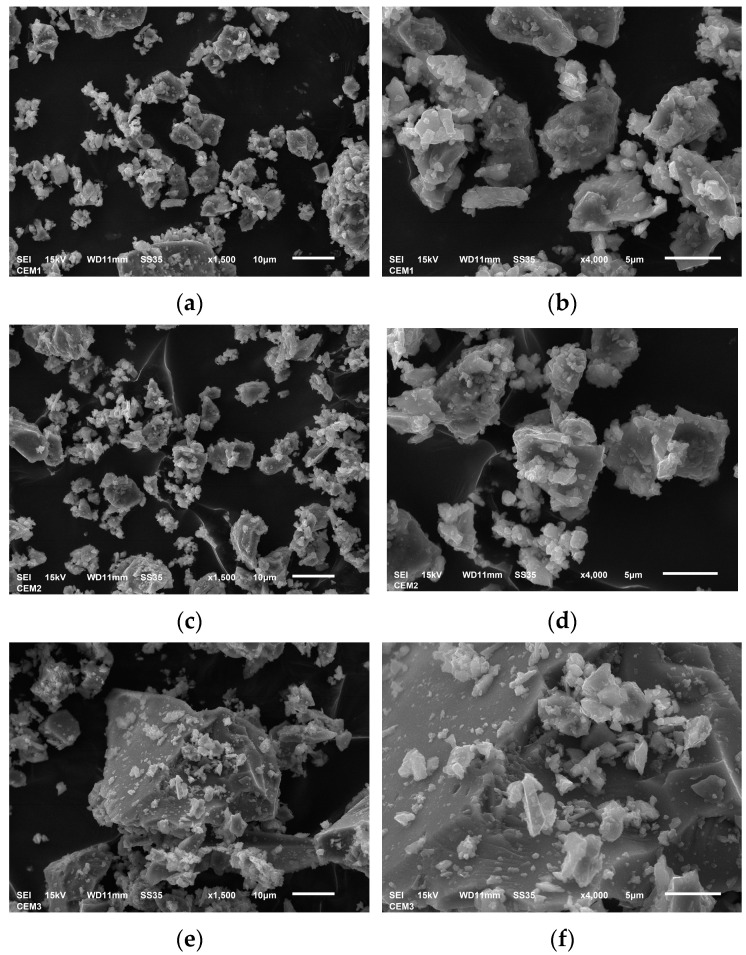
Cement images taken with Jeol JSM-6610 (Joel, Tokyo, Japan): (**a**) CEM I × 1500, (**b**) CEM I × 4000, (**c**) CEM II/B-V × 1500, (**d**) CEM II/A-V × 4000, (**e**) CEM III/A × 1500, and (**f**) CEM III/A × 4000.

Analysis of the cements used was performed using a JSM-6610 scanning microscope (Joel, Tokyo, Japan), in which the samples were applied to carbon tape and dusted with graphite. Analysis of the chemical composition of the tested materials was performed in an EDS analyzer, which is part of the device. Cement images are shown in Figure 1.

**Figure 2:**
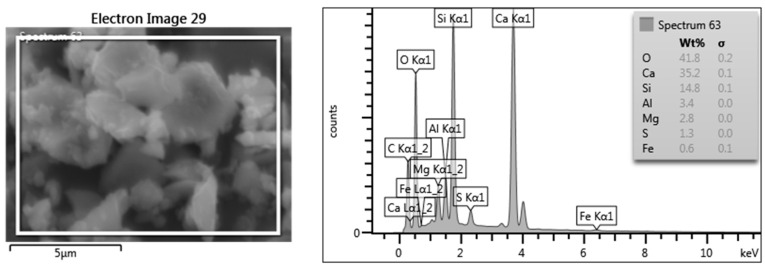
An example of a chemical analysis image for CEM III cement with 45% slag content created using the EDS analysis method.

Cement images were taken with a Jeol JSM-6610 microscope (Jeol, Tokyo, Japan) for CEM I, CEM II, and CEM III cements at magnifications of ×1500 and ×4000. Images a and b for cement CEM I show a compact clinker structure with small amounts of gypsum, which is a setting regulator. In images c and d for CEM II, ash particles can be seen, and in images e and f for CEM III, there is a high content of blast furnace slag. Additionally, chemical analysis was performed for each of the four cements (Table 2):

The results of the analysis of the cement used with a 45% share of blast furnace slag (S) are marked as CEM III/A 42.5 HSR-NA (Figure 2). The obtained results are as follows: CaO—35.2%, Si—14.8%, Al—3.4%, Mg—2.8%, S—1.3%, and Fe—0.6% [36].

### 2.2. Analysis of Grain Size and Reactivity of Aggregates

**Figure 3:**
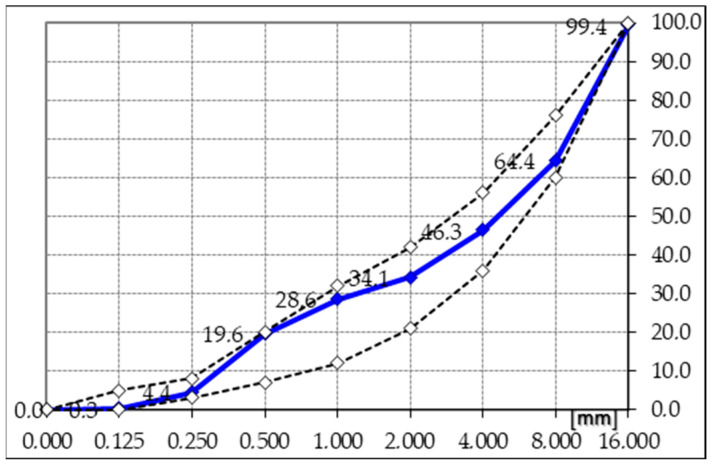
Graining curve of the mineral mixture.

According to the requirements of technical specifications, the aggregate should be characterized by high technical parameters and meet the condition of maximum saturation [39]. The screening analysis of the granite aggregate of 2/8 and 8/16 mm and sand of 0/2 mm is given in Table 3 and Figure 3.

The designed mineral mixture composition and the required specification curves for good grain size are shown in Figure 3.

Due to the requirement of using mineral materials characterized by the lack of a potential alkali–silica reaction (ASR), the potential reactivity of the aggregate was determined using two methods: PB1 (14-day test) and PB2 (365-day test). This determination involved measuring the elongation of the prepared mortar and concrete samples in accordance with the procedure. The final test result, which is presented in Table 4 and Table 5, was in reactivity category R0 for both fine and coarse aggregate in 1 M NaOH solution at 80 °C. The detailed description of the test procedure and analysis of the results in this regard were discussed in more detail in [40]. Table 4 and Table 5 describe the terms resulting from the procedure and are described as L_7_, L_28_, and L_91_, which represent the linear changes in sample length during testing after 7, 28, and 91 days of testing.

The aggregate test results obtained, performed in accordance with procedures PB1 and PB2, allowed the aggregates to be classified as R0, i.e., non-reactive.

After obtaining positive aggregate test results, the formulation compositions shown in Table 6 were designed.

In the described work, over 250 samples were prepared and tested in accordance with the requirements of the PN-EN 12390-2 standard [41]. The scope of individual tests and reference to the requirements for concrete intended for road construction are included in Section 2.3.

### 2.3. Methods

The first phase of the project concerned the testing of hardened concrete and consisted of determining the compressive, bending, and splitting strength after 28, 56, and 90 days [42,43,44]. The density of concrete was determined in accordance with the PN-EN 12390-7 standard [45]. The second phase consisted of determining the durability of concrete with a reduced carbon footprint by testing frost resistance after 150 cycles in accordance with the PN-B-06250:1988 standard [46]. The F150 durability test was conducted on 48 samples with dimensions of 100 × 100 × 100 mm. A total of 144 samples were prepared and tested to determine the strength properties. The starting date of the tests was 28 days for CEM I, 56 days for CEM II, and 90 days for CEM III and CEM V, i.e., at the equivalent time [39,47]. A positive result of this test as specified in the standard must not exceed a 5% loss in mass and a 20% decrease in compressive strength compared to the reference samples. Furthermore, the samples after freeze/thaw cycles must not show cracks. Furthermore, in order to check the durability, resistance to de-icing salts [48], and penetration of water under pressure for each series, the pore distribution in the hardened concrete was determined in accordance with PN-EN 480-11 [49]. The requirements for cement concrete [39,47] used for road construction are presented below in Table 7.

## 3. Results

### 3.1. Results of Assessing the Properties of the Concrete Mixture after 5 and 45 min

The properties of the concrete mixture were determined 5 and 45 min after mixing the ingredients (Table 8). These time intervals were used because experience shows that 45 min is the maximum time for incorporating the mixture without significantly changing its properties and ingredients under normal conditions.

### 3.2. Determination of the Strength Characteristics of Concrete for Road Surfaces

In the first phase of the tests, strength characteristics were determined, such as compressive strength after 28, 56, and 90 days in accordance with PN-EN 12390-3 [42]. Additionally, flexural strength and tensile splitting strength tests of the test specimens were performed in accordance with the PN-EN 12390-5 [43] and PN-EN 12390-6 [44] standards. All samples were prepared and stored in accordance with the EN 12390-2 standard [41]. The average test results of the 144 samples are shown in Table 9.

The highest compressive strength results were obtained after 90 days for the C30/37_III formulation of 70.9 MPa, i.e., 18% more than for the C30/37_I base formulation, for which the result was 58.4 MPa. When assessing the flexural strength for 36 samples, it should be emphasized that for all recipes, the required result of >4.5 MPa required in the technical specification was achieved. The highest results were obtained for the C30/37_III and C30/37_V formulations, which were 6.4 MPa and 6.6 MPa. The last determination was the tensile splitting strength, and here, the highest results were also for the C30/37_III and C30/37_V recipes, which were 5.0 MPa and 5.2 MPa, respectively. It should be emphasized that the obtained results of strength tests for concretes with a reduced carbon footprint met the high requirements for concrete surfaces of roads with low and very high traffic intensity, KR4-KR7.

### 3.3. Testing the Durability of Road Concrete Using Multi-Component Cements

An assessment of the possibility of using concrete with a reduced carbon footprint made of low-emission cements is possible after performing comprehensive durability tests. As part of the durability assessment, 150 freezing and thawing cycles were performed to determine frost resistance, resistance to de-icing salts was checked, the depth of water penetration under pressure was determined, and the air entrainment structure in the concrete was determined as a final verification of durability.

#### 3.3.1. Frost Resistance after 150 Cycles

The determination of frost resistance in cyclic freezing and thawing at temperatures −20 °C and +20 °C was performed in accordance with the PN-B-06250:1988 standard [46]. As part of the study, 12 cubic samples with dimensions of 100 × 100 × 100 mm were prepared for each recipe. After 150 test cycles, the % loss of compressive strength and weight loss were determined. Samples after 150 freezing and thawing cycles must not show cracks. The average results from the four tests are shown in Table 10.

#### 3.3.2. Resistance to Deicing Salts According to EN 12390 PKN-CEN/TS 12390-9

The test was performed in accordance with the EN 12390 PKN-CEN/TS 12390-9 standard [48] and involved the cyclic freezing and thawing of concrete samples in the presence of de-icing salts (3% NaCl). After 28 and 56 days, the mass loss of the samples was determined, and the determined loss must be less than 2. A positive test result is when, for each sample, the mass loss was less than 1.5 kg/m^2^, and the average mass loss was determined after 28 and 56 days. 

#### 3.3.3. Air Void Analysis

**Figure 4:**
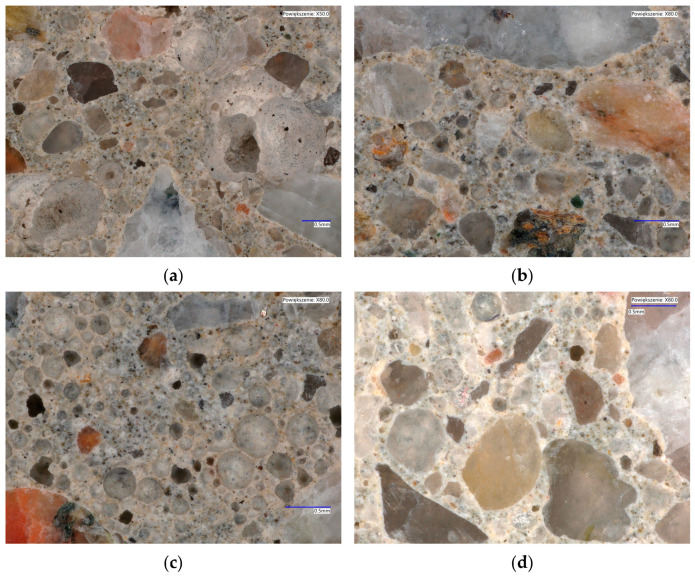
Images of the structure of prepared concrete samples: (**a**) C30/37_I, (**b**) C30/37_II, (**c**) C30/37_III, and (**d**) C30/37_V.

The determination of the air-entrainment structure in concrete was performed in accordance with the procedure described in the PN-EN 480-11:2008 standard [49]. Two samples were cut from the solid samples for analysis, with dimensions of 150 × 100 × 20 mm. After grinding the samples, they were polished, and contrast was added to obtain better accuracy. Automatic analysis of the number and size of air voids was performed using the microscopic method using the Nikon SMZ1270 Navitar computer automatic image analysis system (Tokyo, Japan). The image and average values from four measurements for each sample are shown in Figure 4 and Table 10.

A very effective and commonly used method for assessing the durability of cement concrete over time is to assess the structure of the void distribution *L* (the distance between the voids), which should be less than 0.2 mm. The second assessment parameter is the content of micropores with a diameter of less than 0.3 mm, described as *A*_300_, which must be greater than 1.5%. Additionally, the total amount of air in the concrete was determined (*A*). The results of average measurements for each of the four samples are presented in accordance with the standard [49] in Table 10. The total air content *A* is comparable in value to the results for the concrete mixture before forming (from 4.9% to 6.2%) because the air content in hardened concrete is 4.9 to 6.0%. The most favorable results were obtained for the C30/37_V recipe based on CEM V cement.

The results of frost resistance determination after 150 cycles indicate that the smallest decrease in compressive strength was obtained for the C30/37_III recipe on CEM III cement. This decrease was 0.9% and the decrease in sample weight was only 0.1%. In terms of determining resistance to deicing agents (salt), the most favorable results were obtained for the C30/37_V and C30/37_III formulas and were in line with the requirements of the standard. As part of the durability tests, water penetration tests were also carried out under pressure, and for all designed compositions, the results ranged from 17 to 24 mm, where the requirement was < 45 mm. The obtained results of the analysis of the air structure in hardened concrete are very similar to the results obtained using the pressure method for the concrete mix, which may indicate the appropriate amount of air-entraining admixture and ranges from 4.9% to 6.2%. The total air content in hardened concrete is in the range of 4.9–6.0%. By analyzing the results obtained to determine the characteristics of the spacing of air voids, it can be concluded that they meet the requirements for concrete used in XF conditions.

### 3.4. Carbon Footprint Reduction

The aim of the study was to determine the possibility of reducing the carbon footprint in concrete by using multi-component cements. These cements differed in the amount of clinker and the type and amount of mineral additives in their composition. For calculations using the LCA (Life Cycle Assessment) method according to the ISO 14067 standard [50], the actual transport distances of cement and aggregate to the construction site were assumed, which were 100 and 120 km. Having calculated CO_2_ emissions for individual components of the concrete mix and knowing the distance from the construction site, the determined carbon footprint is presented in Table 11.

Analyzing the results obtained using the software recommended by the Global Cement and Concrete Association (GCCA), it can be seen that the use of CEM II cement allows a reduction in the carbon footprint of 17.4%, and the use of CEM V and CEM III cement in concrete intended for the construction of roads, engineering structures, and airports allowed a reduction of up to 35.9 and 39.4%. This is an important result and suggests greater use of cements with mineral additives for infrastructure construction should be considered.

## 4. Discussion

The CEM III and CEM V multi-component cements used in road concrete were characterized by the highest compressive strength, above 70 MPa, obtained for CEM III concrete and 68 MPa for CEM V concrete. However, CEM I cement, commonly used in infrastructure, achieved the lowest compressive strength of 58 MPa. These results confirmed the usefulness of multi-component cements in durability tests, as the smallest decrease in compressive strength after 150 freeze–thaw cycles was noted for CEM III (−0.9%) and CEM V (−1.4%) concretes. This can be confirmed by the results of water penetration under pressure, because for the recipes using CEM II, CEM III, and CEM V, 17, 18, and 17 mm of water penetration were obtained, which proves the high tightness of the concrete. The final verification took place during the analysis of the aeration structure performed under a microscope in accordance with the requirements of the PN-EN 480-11 standard [49]. The analysis results confirmed the proper structure and minimal spacing of air bubbles in the concrete, which guarantees durability. Concrete using CEM II, CEM III, and CEM V can be used to build local roads and even expressways with heavy vehicle traffic. In addition, the concrete mix based on CEM V is characterized by a carbon footprint reduced by 36%, and the mix based on CEM III by a reduction of up to 39% for the A1–A3 phase. Carbon footprint calculations were performed for the processes of extraction and transport of raw materials and the production of concrete at the plant. Considering that this A1–A3 phase could be responsible for a reduction in the carbon footprint of up to 40%, such concrete can be classified as environmentally friendly concrete, consistent with the idea of sustainable development of modern building materials. Surface concrete designed with the addition of low-clinker cements such as CEM II, CEM III, and CEM V meets the very high requirements for a modern concrete surface intended for the construction of local roads and parking lots. The results of tests on the durability of road concrete clearly indicate that concrete with a reduced carbon footprint is a sustainable solution and an alternative to the commonly used and required Portland cement CEM I 42.5 in Poland. The cements used are blast furnace slag, silica fly ash, and limestone, which are waste materials, thanks to which we can reduce our carbon footprint and reduce CO_2_ emissions by over 40%. Thanks to this research, concrete with a reduced carbon footprint can be successfully used for the construction of roads and communication infrastructure, in line with the sustainable construction strategy.

## 5. Conclusions

The obtained test results for road concrete with a reduced carbon footprint thanks to the use of low-clinker cements allow the following conclusions to be drawn:

1. Road concrete designed with CEM V is characterized by a reduced carbon footprint of 36%, and for a mixture based on CEM III, as much as 39%, which is why such concrete can be classified as environmentally friendly concrete and materials consistent with the sustainable development of modern construction.
2. The highest compressive strength exceeding 70 MPa was obtained for the C30/37_III concrete formulation, while 68 MPa was observed for the C30/37_V concrete, and the lowest value was observed for the C30/37_I comparative formulation after 28, 56, and 90 days.
3. The most favorable results of frost resistance in cyclic freezing and thawing were obtained for the recipe using CEM III (−0.9%) and CEM V (−1.4%) cement. This is confirmed by the results of water penetration under pressure, because for the recipes using CEM II, CEM III, and CEM V, water penetration of 17, 18, and 17 mm was obtained, which proves the high tightness of the concrete.
4. Concrete using CEM II, CEM III, and CEM V can be used to build local roads and even expressways with heavy vehicle traffic.
5. The microscopic analysis of the pore structure in the hardened concrete allows us to conclude that the total air content is similar to the results obtained in the concrete mix using the pressure method, ranging from 4.9% to 6.2%. The total air content in hardened concrete ranges from 4.9 to 6.0%. The highest value of the air permeability coefficient (*L*) and the lowest micropore content (*A*_300_) was demonstrated by concrete marked as C30/37_V based on CEM V cement.

## Figures and Tables

**Table 1:** Properties of cements.

Property	Unit	CEM I 42.5 R	CEM II A-V 42.5 R	CEM III A 42.5 N-LH/HSR/NA	CEM V A S-V 42.5 N-LH/HSR/NA
chemical composition of cement					
CaO	%	62.82	55.44	50.54	46.3
SiO_2_	%	18.95	23.58	29.18	28.7
Al_2_O_3_	%	4.99	7.83	6.17	9.7
SO_3_	%	3.14	2.83	2.48	2.74
Fe_2_O_3_	%	2.81	3.25	1.55	3.0
MgO	%	1.37	1.56	4.04	2.06
K_2_O	%	0.88	0.71	0.69	1.08
eqNa_2_O	%	0.79	0.73	0.78	1.06
Na_2_O	%	0.21	0.26	0.33	0.35
Cl^−^	%	0.08	0.076	0.07	0.041
physical and mechanical properties of cement					
Compressive strength 2 days	MPa	27.8	26.1	14.2	20.0
Compressive strength 28 days	MPa	56.7	53.4	51.5	58.1
Specific surface	cm^2^/g	3374	3716	4466	4800
Beginning of cement setting	minutes	208	179	238	275
End of cement setting	minutes	284	260	317	350
Consistency in volume	mm	0.8	0.88	0.8	0.81
Water demand of cement	%	27.8	27.5	29.8	32.2

**Table 2:** The chemical analysis of the composition of CEM I, CEM II, CEM III, and CEM V.

Element	CEM I 42.5 R	CEM II A-V 42.5 R	CEM III A 42.5 N-LH/HSR/NA	CEM V A S-V 42.5 N-LH/HSR/NA	Standard Label
	wt%	wt%	wt%	wt%	
O	37.92	39.61	41.8	38.54	SiO_2_
Mg	1.06	0.56	2.8	0.97	MgO
Al	3.59	1.37	3.4	3.14	Al_2_O_3_
Si	9.15	9.09	14.8	9.87	SiO_2_
K	0.52	1.41	1.4	1.56	KBr
Ca	44.68	46.26	35.2	44.26	Wollastonite
Fe	3.07	1.70	0.6	1.66	Fe
Total:	100.00	100.00	100.00	100.00	

**Table 3:** Graining of materials.

Sieve [mm]	Volume of Aggregate on Individual Control Sieves [%]		
	Granite 8/16	Granite 2/8	Sand 0/2
16	1.7	0.0	0.0
8	96.3	1.2	0.0
4	2.0	59.7	0.0
2	0.0	39.1	2.5
1	0.0	0.0	15.9
0.5	0.0	0.0	25.5
0.25	0.0	0.0	43.4
0.125	0.0	0.0	11.9
0.0	0.0	0.0	0.8

**Table 4:** Determination of the ASR for 0/2mm aggregate.

Samples	Change in Length on Individual Days [mm]					
	L_7_	L_14_	L_28_	L_91_	L_182_	L_365_
1	−0.002	0.003	0.001	0.008	0.014	0.016
2	−0.004	0.003	0.001	0.009	0.012	0.015
3	−0.003	0.003	0.002	0.007	0.012	0.014
Average value	−0.003	0.003	0.001	0.008	0.013	0.015

**Table 5:** Determination of the ASR for 2/8 and 8/16 mm aggregate.

Samples	Change in Length on Individual Days [mm]					
	L_7_	L_14_	L_28_	L_91_	L_182_	L_365_
1	0.000	−0.001	−0.002	0.006	0.018	0.027
2	−0.001	0.000	−0.003	0.006	0.016	0.019
3	−0.001	−0.001	−0.001	0.006	0.016	0.022
Average value	−0.001	−0.001	−0.002	0.006	0.017	0.023

**Table 6:** Designed composition of concrete mixtures [kg/m^3^].

Materials	Concrete Mix Compositions [kg/m^3^]			
	C30/37_I	C30/37_II	C30/37_III	C30/37_V
CEM I 42.5 R	360	-	-	-
CEM II/A-V 42.5 R	-	360	-	-
CEM III A 42.5 HSR/NA	-	-	360	-
CEM V/A S-V 42.5 N-LH/HSR/NA	-	-	-	360
Water	144	144	144	144
Fine aggregate sand 0/2 mm	682	682	682	682
Coarse granite aggregate 2/8 mm	565	565	565	565
Coarse granite aggregate 8/16 mm	702	702	702	702
SP PC	2.5	2.5	2.5	2.5
LPA	0.7	0.7	0.7	0.7
Density	2456	2450	2452	2449

**Table 7:** Requirements for the concrete pavement for traffic categories KR1 ÷ KR4.

Properties of Pavement Concrete	Requirements	Test Method
Compressive strength	C30/37	PN-EN 12390-3 [42]
Frost resistance F150:		PN-B-06250 [46]
-Loss of sample mass	<5%	
-Loss of compressive strength	<20%	
Characteristics of air voids in concrete:		PN-EN 480-11 [49]
-content of micropores with a diameter of less than 0.3 mm (*A*_300_), %	≥1.5%	
-index of pore distribution in concrete, *L* mm	≤0.200 mm	
Density, tolerance in reference to the formula	±3.0%	PN-EN 12390-7 [45]

**Table 8:** Properties of the concrete mix.

Materials	Concrete Mix Compositions [kg/m^3^]			
	C30/37_I	C30/37_II	C30/37_III	C30/37_V
Consistency after 5 min, mm	120	110	140	140
Consistency after 45 min, mm	80	95	130	125
Air content after 5 min, %	5.5	5.6	5.7	6.2
Air content after 45 min, %	4.9	5.2	5.6	6.0
Density, g/cm^3^	2.456	2.452	2.454	2.448

**Table 9:** Strength properties of road concrete.

C30/37 Road Concrete	Study Start Date [MPa]		
	28 Day	56 Day	90 Day
	Compressive strength		
C30/37_I with CEM I 42.5 R	54.5 ± 2.1	55.6 ± 1.8	58.4 ± 3.1
C30/37_II with CEM II/A-V 42.5 R	48.4 ± 1.8	56.1 ± 1.9	59.3 ± 1.3
C30/37_III with CEM III/A 42.5 N-LH/HSR/NA	43.2 ± 3.2	60.7 ± 2.1	70.9 ± 2.2
C30/37_V with CEM V/A S-V 42.5 N-LH/HSR/NA	42.3 ± 1.1	54.6 ± 1.1	68.6 ± 0.9
	Flexural strength		
C30/37_I with CEM I 42.5 R	5.2 ± 0.9	5.4 ± 0.7	5.3 ± 0.4
C30/37_II with CEM II/A-V 42.5 R	4.8 ± 0.5	5.2 ± 1.1	5.5 ± 0.8
C30/37_III with CEM III/A 42.5 N-LH/HSR/NA	4.6 ± 1.2	5.5 ± 0.9	6.4 ± 0.9
C30/37_V with CEM V/A S-V 42.5 N-LH/HSR/NA	4.5 ± 0.4	5.3 ± 0.6	6.6 ± 1.1
	Tensile Splitting Strength of the Test Specimens		
C30/37_I with CEM I 42.5 R	4.0 ± 0.5	4.3 ± 0.2	4.2 ± 0.8
C30/37_II with CEM II/A-V 42.5 R	4.1 ± 0.2	4.4 ± 0.8	4.7 ± 1.0
C30/37_III with CEM III/A 42.5 N-LH/HSR/NA	3.7 ± 0.7	4.3 ± 0.5	5.0 ± 0.5
C30/37_V with CEM V/A S-V 42.5 N-LH/HSR/NA	3.6 ± 0.3	4.1 ± 0.4	5.2 ± 0.3

**Table 10:** The influence of low-clinker cements on the durability of road concrete.

Materials	Concrete Mix Compositions [kg/m^3^]				
	C30/37_I	C30/37_II	C30/37_III	C30/37_V	Requirement
	Frost Resistance Test F150 average result				
Loss of compressive strength ΔR, %	−4.5	−2.2	−0.9	−1.4	<20%
Weight loss of samples ΔG, %	−0.2	−0.12	−0.1	−0.1	<5%
	Degree of Defect m_56_/m_28_ average result				
Resistance to frost in the presence of de-icing salt	1.22	0.95	0.77	0.82	<2
	Water penetration under pressure				
Depth of water penetration under pressure, mm	24	17	18	17	<45
Characteristics of air voids in hardened concrete					
Total air content, *A*, %	4.9	5.2	5.6	6.0	4.5–6.0
Spacing factor, *L*, mm	0.18	0.17	0.14	0.13	<0.20
Micro air-void content*, A*_300_*,* %	2.10	1.85	2.45	2.50	>1.50

**Table 11:** Carbon footprint reduction results of paving concrete with multi-component cements [9,11].

Parameter	Unit	C30/37_I	C30/37_II	C30/37_III	C30/37_V
Carbon footprint cement	CO_2_/kg	0.703	0.559	0.377	0.405
Cement transportation	CO_2_/km/t	0.199	0.199	0.199	0.199
Carbon footprint granite aggregate	CO_2_/kg	0.007	0.007	0.007	0.007
Aggregate transport	CO_2_/km/t	0.166	0.166	0.166	0.166
The carbon footprint of concrete in cubic meters	CO_2_/m^3^	**298**	**247**	**181**	**191**
Carbon footprint reduction	[%]	0.0	−17.4	−39.4	−35.9

## Data Availability

The original contributions presented in the study are included in the article, further inquiries can be directed to the corresponding author.
